# The implementation of a smoking cessation and alcohol abstinence intervention for people experiencing homelessness

**DOI:** 10.1186/s12889-022-13563-5

**Published:** 2022-06-27

**Authors:** Rebekah Pratt, Serena Xiong, Azul Kmiecik, Cathy Strobel-Ayres, Anne Joseph, Susan A. Everson Rose, Xianghua Luo, Ned Cooney, Janet Thomas, Shelia Specker, Kola Okuyemi

**Affiliations:** 1 Department of Family Medicine and Community Health, Program in Health Disparities Research, University of Minnesota, 717 Delaware Street, Minneapolis, MN 55414 USA; 2 Department of Family Medicine and Community Health, University of Minnesota, 717 Delaware Street, Minneapolis, MN 55414 USA; 3 Department of Medicine, University of Minnesota, 401 East River Parkway, Minneapolis, MN 55455 USA; 4 Department of Medicine & Program in Health Disparities Research, University of Minnesota, Minneapolis, USA; 5 Division of Biostatistics, School of Public Health and Masonic Cancer Center, University of Minnesota, 420 Delaware Street SE, MMC 303, Minneapolis, MN 55455 USA; 6 Department of Psychiatry, Yale University School of Medicine, 300 George Street #901, New Haven, CT 06511 USA; 7 Department of Medicine, Division of General Internal Medicine, Program in Health Disparities Research, University of Minnesota, 717 Delaware Street, Suite 166, Minneapolis, MN 55414 USA; 8 Department of Psychiatry, University of Minnesota, 2312 S 6th Street, Minneapolis, MN 55454 USA; 9 Department of Family & Preventive Medicine, University of Utah, 375 Chipeta Way, Suite A, Salt Lake City, UT 84108 USA

## Abstract

**Background:**

In the United States, eighty percent of the adult homeless population smokes cigarettes compared to 15 percent of the general population. In 2017 Power to Quit 2 (PTQ2), a randomized clinical trial, was implemented in two urban homeless shelters in the Upper Midwest to address concurrent smoking cessation and alcohol treatment among people experiencing homelessness. A subset of this study population were interviewed to assess their experiences of study intervention. The objective of this study was to use participants’ experiences with the intervention to inform future implementation efforts of combined smoking cessation and alcohol abstinence interventions, guided by the Consolidated Framework for Implementation Research (CFIR).

**Methods:**

Qualitative semi-structured interviews were conducted with 40 PTQ2 participants between 2016–2017 and analyzed in 2019. Interviews were audio-recorded, transcribed, and analyzed using a socially constructivist approach to grounded theory.

**Results:**

Participants described the PTQ2 intervention in positive terms. Participants valued the opportunity to obtain both counseling and nicotine-replacement therapy products (intervention characteristics) and described forming a bond with the PTQ2 staff and reliance on them for emotional support and encouragement (characteristics of individuals). However, the culture of alcohol use and cigarette smoking around the shelter environment presented a serious challenge (outer setting). The study setting and the multiple competing needs of participants were reported as the most challenging barriers to implementation (implementation process).

**Conclusion:**

There are unique challenges in addressing smoking cessation with people experiencing homelessness. For those in shelters there can be the difficulty of pro-smoking norms in and around the shelter itself. Considering pairing cessation with policy level interventions targeting smoke-free spaces, or pairing cessation with housing support efforts may be worthwhile.. Participants described a discord in their personal goals of reduction compared with the study goals of complete abstinence, which may pose a challenge to the ways in which success is defined for people experiencing homelessness.

**Trial registration:**

Clinicaltrials.gov, NCT01932996, registered 08/30/2013.

**Supplementary Information:**

The online version contains supplementary material available at 10.1186/s12889-022-13563-5.

## Background

Approximately 1.5% of adults living in the United States experience homelessness annually and up to 4.2% of adults living in the United States will experience homelessness in their lifetime [1]. Homelessness presents a unique set of challenges that can negatively impact health [2] and presents an important public health concern. Eighty percent of the adult homeless population smokes cigarettes [3] compared to 15 percent of the general population [4], therefore determining ways to engage this community in smoking cessation is crucial to mitigating the impact of homelessness on preventable mortality and morbidity [4]. While smokers experiencing homelessness report interest in smoking cessation [5–8], there are multiple competing priorities and barriers demands, in particular, concerns about the social environment and daily stress [7, 9–11]. Cessation intervention efforts to date have resulted in minimal quit rates [4, 12].

There is a paucity of information on the processes involved in implementing smoking cessation interventions among people experiencing homelessness. Implementation Science (IS) studies the process of intervention implementation [13] and may offer a valuable perspective in better understanding how cessation approaches could best be implemented for people experiencing homelessness. The Consolidated Framework for Implementation Research (CFIR) [14–16] has been widely used in health services research [15] and focuses on five key areas of implementation.

The first CFIR domain focuses on the intervention characteristics, including the perceived strength and quality of the intervention, the relative advantage and adaptability of the intervention and the source of the intervention content [14]. The second domain is termed the inner setting, which considers culture and climate and the fit between individual participant values and the intervention content [14]. The third domain is the outer setting, which focuses on patient needs and resources, peer pressure and the broader policy context in which the intervention is delivered [14]. The fourth domain pertains to the characteristics of the individual participant including self-efficacy, knowledge, beliefs, and readiness to change [14]. Finally, the fifth domain focuses on the implementation process, such as the role of engagement and evaluation [14]. CFIR has been applied to the field of smoking cessation [17, 18] and substance use disorders [19, 20], but has not been utilized to understand the unique characteristics of smoking behavior change among those living with homelessness.

Overall, there is little research published that would inform the CFIR domains and smoking cessation for people experiencing homelessness. There is some literature that helps to inform the third domain of the outer setting, in particular the impact of pro-smoking norms commonly found in shelter environments [8, 21, 22], including high rates of smoking among people frequenting shelters [6, 11], making it particularly challenging to quit [8, 10, 21]. Alternately, stable housing has been positively associated with abstinence outcomes [22, 23], although shelters may offer access to supportive health services [24] to help with addressing smoking.

The majority of the literature published focuses on the fourth CFIR domain, the characteristics of individuals utilizing a smoking cessation intervention, and these have identified psychosocial variables such as shame and stigma around smoking [25, 26]. Additionally, there is high prevalence of concurrent tobacco and alcohol use among people experiencing homelessness [27], and it may be beneficial to address these two behaviors simultaneously [28, 29].

Studies that have targeted smoking cessation among smokers with alcohol use disorders and findings show an average 7 percent quit rate, and high rates of relapse [30]. Some evidence suggests that addressing smoking can improve alcohol abstinence [31], although studies show mixed results [32, 33].

Power to Quit 2 (PTQ2) was a randomized controlled trial, built on the findings from the first PTQ study, aimed to investigate concurrent smoking cessation and alcohol treatment among people experiencing homelessness [34, 35]. In this study, we present findings from semi-structured interviews with participants completing PTQ2. The study aim was to explore the experience of participating in a smoking and alcohol intervention, and to provide insight into the challenges faced by participants when trying to quit smoking. Additionally, the analysis drew on the CFIR framework [14] to inform future learning on the intervention implementation process.

## Methods

PTQ2 was a randomized clinical trial focusing on tobacco and alcohol use that used a three-group design that included (1) Usual care (UC) for smoking and alcohol cessation (control group), (2) Intensive smoking cessation plus UC alcohol abstinence counseling (IS), and (3) Integrated Intensive Smoking and Intensive Alcohol Counseling (IntS + A). The counselling was a cognitive behavioral therapy approach to smoking cessation and alcohol abstinence, and conducted as individual sessions. All participants received 12 weeks of nicotine replacement therapy, with nicotine patches (tailored to their baseline cigarettes smoked per day), plus their choice of nicotine gum or lozenge. A full explanation of the design and methods can be found elsewhere [11, 34, 35]. During the RCT consent process, PTQ2 participants were informed that they might be invited to participate in an interview portion of the study. Research study staff approached potential participants just prior to the final study visit (week 26). In recognition of their time and effort, participants were compensated with a $20 gift card, paid for by the research grant funds.

### Study population

A convenience sample of 40 PTQ2 participants was recruited to participate in sharing their experience of the study. Interviews were conducted with 25 intervention (IS, IntS + A) and 15 control group participants. The eligibility criteria was that participants had concluded participation in the study intervention or control study conditions, within four weeks of the interview. Control arm participants were recruited with the intention of ensuring that participation in the interviews did not have any disproportionate impact on study participant experience or outcomes.

### Study instrument

The research study team developed the semi-structured interview guide (see Additional file 1) for this study with a goal of collecting data on the implementation of the study from the perspective of the participants [11]. The interview guide explored participants’ experience of attempting to quit smoking during the study, their experience with the study intervention, and their overall views on participating in research. Sample questions, which were informed by the CFIR model, included: “You mentioned you received (education/sessions on smoking/sessions on smoking and alcohol) as part of the study. What was your overall impression of doing these activities?”, “Did the sessions have any impact on your (smoking or smoking and drinking)?”, “How did you feel about the amount of education or counselling you received?” and, “In general, do you have any views on how dealing with homelessness impacts the ability of people to take part in studies like this?” Interviews lasted from 20 to 60 min in length. The Alcohol Use Disorder Identification Test (AUDIT) [36], a 10-item scale that measures drinking behavior, dependence, and consequences related to drinking, was used to measure alcohol use severity.

### Data collection

Semi-structured interviews were conducted in-person between December 2016 and April 2017. In order to avoid bias responses to questions regarding the study and the study team, a Masters in Public Health trained, non-study staff member (AK) conducted the interviews. Interviews were conducted in two of the urban shelters where the study team was delivering the intervention, and were conducted in a private space with the interviewer and interviewee. One interview was conducted with two interviewees together with the interviewer.

### Data analysis

Interviews were audio recorded, transcribed verbatim and the qualitative data were analyzed in 2019 using NVivo 12 [37]. Three members of the research team coded the transcripts (RP, AK and GR), and double coded a sub-set of data. Training on the analytic process was provided by the lead coder (RP). The research team used the social constructivist approach to grounded theory to identify themes and sub-themes in the data [38, 39]. While grounded theory often allows for themes to emerge from the analysis without consideration of additional factors such as the literature, the socially constructivist version of grounded theory developed by Charmaz allows for themes to both emerge from the data, and be reviewed in relation to existing literature or theoretical frameworks, such as CFIR. Discussions with all members of the research team on the emerging analysis were held throughout the analysis to help ensure the rigor of the qualitative analysis. These discussions also included time and space to engage in reflexivity on the various experiences and identities of the research team members in comparison to those of the study participants. The study team included people who had lived experience of homelessness, and a consensus building approach was used to integrate any differences in the emerging analysis, and draw on the strengths of the different identities of team members in interpreting the analysis. The analysis focused on the experience of the study implementation, additional analyses of the participant’s experience of the social and environmental influences on smoking is reported elsewhere [11].

### Human subjects

The University of Minnesota Institutional Review Board provided ethical approval for the conduct of this study.

## Results

We present participant demographics, followed by key findings from the interviews in relation to overarching CFIR domains (*Intervention Characteristics, Outer Setting, Inner Setting, Characteristics of Individuals, Implementation Process).*

### Demographics

A subset of participants were recruited from the main study population of 432. Baseline demographic characteristics of the subset of participants from the RCT who participated in the interviews are shown in Table 1, [11] and were broadly reflective of the main study demographics. Thirty-two participants identified as African American/Black, six as White, one as Native American/Alaska Native, and one as more than one race. Eleven participants were female, and 29 were male. Housing stability was assessed by self-report on a scale of 0 (not at all stable) to 10 (extremely stable) and the mean (± SD) response was 3.53 ± 3.48 (range, 0 to 10). Most participants identified themselves as unemployed. Participants smoked on average 14.6 ± 8.3 (range 2.5 to 40) cigarettes per day at their eligibility screening and just over half had their first cigarette of the day within 30 min of waking. Participant AUDIT scores averaged 14.9 ± 4.87 (range 7 to 24) which corresponds to risky/hazardous or high-risk/harmful alcohol use risk levels.

**Table 1:** Participant baseline demographics and characteristics

	Mean ± SD (range) or n (%)
N	40
Study randomization arm	
A: Standard Care	15 (37.5%)
B: Intensive Smoking Intervention	13 (32.5%)
C: Intensive Smoking and Alcohol Intervention	12 (30.0%)
Age	50.20 ± 9.2 (29.6–69.5)
Sex	
Male	29 (72.5%)
Female	11 (27.5%)
Cigarettes smoked per day (on eligibility survey)^a^	14.6 ± 8.3 (2.5–40)
Housing situation (at eligibility survey)	
Emergency or overnight shelter	23 (57.5%)
Campsite, vehicle, abandoned building/house, parking garage, or on the street	7 (17.5%)
Transitional or supportive housing, long-term shelter	5 (12.5%)
Staying with relative, friend, or other people/double-up – less than 3 months at the same place	5 (12.5%)
Housing stability (self-rating from 0-not at all stable to 10-extremely stable)	3.53 ± 3.48 (0–10)
Race	
African American or Black	32 (80.0%)
Native American/Alaskan Native	1 (2.50%)
White	6 (15.0%)
More than 1 race	1 (2.5%)
Education	
Some high school or less	12 (30.0%)
High school graduate or GED	14 (35.0%)
Some college or technical school	13 (32.5%)
Unknown/not reported	1 (2.5%)
Employment	
Employed full time	2 (5.0%)
Employed part time	4 (10.0%)
Out of work for more than 1 year	8 (20.0%)
Out of work for less than 1 year	7 (17.5%)
Unable to work or disabled	19 (47.5%)
Income	
Less than $400 per month	17 (42.5%)
$400-$799 per month	15 (37.5%)
$800-$1,199 per month	6 (15.0%)
$1,200-$1,799 per month	2 (5.0%)
Number of children	2.73 ± 2.21 (0–10)
MINI Psychotic Symptoms Score at Baseline	0.58 ± 1.11 (0–4)
Marijuana use ≥ 20 days in prior 30 days (n, % yes)	3 (7.5%)
Rost-Burnam Screener for Drug Abuse (n, % yes)	37 (92.5%)
Depressive Symptoms (PHQ-9)	7.38 ± 6.36 (0–23)
Perceived Stress (PSS-4)	6.35 ± 3.05 (1–13)
Anxiety (MINI)	2.13 ± 2.95 (0–9)
FTND Minutes to 1^st^ Cigarette	
0–5 min	13 (32.5%)
6–15 min	8 (20.0%)
16–30 min	9 (22.5%)
31–60 min	6 (15.0%)
61 + minutes	4 (10.0%)
Alcohol-Use Severity (AUDIT-10 in Eligibility Survey)	14.93 ± 4.87 (7–24)

^a^n = 4 participants smoked < 5 CPD in the 7 days prior to the eligibility survey, but had missing data for their avg. CPD. For these participants, 2.5 CPD was assumed

### Intervention characteristics

The intervention included a combination of counseling and nicotine replacement therapy (NRT patch plus gum or lozenge) to help manage nicotine withdrawal. Overall the smoking and alcohol cessation intervention was perceived as acceptable by participants. Some participants described appreciating the opportunity to discuss their health, as much of the resource services offered by the shelter focused on addressing their homelessness. Participants who were randomized to the smoking and alcohol counseling arm (vs health education) mentioned the importance of their counselors’ kind demeanor. Having a warm, friendly, and approachable attitude was key to participants’ overall experience in the study.

“Yeah, as far as staff, I never felt like I was less than them just because of being in here at (the shelter). They always made me feel welcome. (Intervention group participant).

Counseling sessions, which became a part of participants’ routine, were described as contributing to an increased sense of purpose. However, while most of the participants saw the benefits of the counseling sessions, some participants were resentful of having to participate in counseling sessions, and felt their counselor was nosy and intrusive. Some participants receiving the one time health education counseling session group believed they would have benefitted from going to the ongoing counseling sessions while others were relieved not to have to attend them. While a few participants believed they would have benefitted from more frequent sessions, for the most part participants were content with the amount of counseling sessions received.

“I liked it the whole session. I didn’t just want the patches, to come and go. I needed the counseling, too” (Intervention group participant).

Overall, participants described that staff provided a comfortable and respectful environment, with the counseling providing a space for focus, reflection, motivation, and skill-building. Occupying one’s time with other, non-smoking activities was a key strategy participants used when they had the urge to smoke or drink. Across the board, participants were educated about the consequences of smoking or drinking on the body and were able to reflect on the ways in which those behaviors were detrimental to their own health. The health consequences of tobacco or alcohol use strongly resonated with participants and the impact of this was present throughout participant responses.

In addition to counselling, participants were also offered Nicotine Replacement Therapy (NRT). NRT for the most part, was described as helping participants manage their cravings. NRT gum and lozenges were reported as being moderately successful in managing craving, with participants being most enthusiastic about having an option for using gum. Most participants shared positive experiences of NRT patches helping to reduce cravings, feeling they had been very helpful. However, some participants reported that the patch did not reduce the urge to smoke.. Some people reported difficulties in keeping the patch adhered to their skin. Physiological cravings were reported to increased appetite and overeating, which subsequently led to weight gain and a fear of putting on weight.

### Outer setting

Participants described a range of factors external to the intervention or intervention setting, such as the broader physical environment, or their own motivation, that impacted their experience of the study. A significant challenge related to the shelter environment was the perception of the ubiquitous use of cigarette smoking and alcohol abuse. In fact, some participants reported having started smoking for the first time since their stay at the shelter. Participants described experiencing frequent temptation and peer pressure to drink and smoke from other shelter residents in areas immediately around the shelter. Smoking and drinking were both described as very important in the social life for shelter residents.

“It’s kind of hard, you’re walking down the street and all of a sudden you’re in a puff of smoke, you’re like wait, I could use one of those” (Intervention group participant).

Getting to counselling sessions was convenient for participants who lived in the shelter; however, for participants who had moved to more stable housing during the study duration, returning to the shelter for appointments was a challenge. Additionally participants described challenges getting to appointments because of adverse weather, conflicts with work, and conflicts with doctor appointments.

The need to find housing, while having very limited financial resources, was another challenge for participants. Cutting back on cigarettes and problem drinking was reported as helping some participants alleviate the financial burden of smoking and/or drinking, cultivating feelings of accomplishment and pride in cutting back their consumption behaviors, and feeling better physically and emotionally.

Many participants described feeling that their personal strength and ability to focus on their goals was what led them to be a part of the study. Participants faced common challenges to smoking cessation, such as dealing with cravings and urges to smoke. Study participants were asked to set a goal of quitting smoking and drinking, however participants frequently identified that they often had their own goal of lessening smoking or drinking, rather than quitting. For many, smoking and drinking were described as habitually intertwined. Engagement in either habit was seen as a trigger spurring engagement in the other. Likewise, reduction or quitting of one, was also associated with the reduction or quitting of the other. Participants reported reductions in smoking or drinking as personal successes.

“I was doing like a couple packs a day, so for me to go from that to six cigarettes a day, that’s like a miracle to me!” (Intervention group participant).

Many participants described forming a bond with the PTQ2 staff and reliance on them for emotional support and encouragement. Many were also glad to have the opportunity to branch out and interact with different people.

### Inner setting

The shelter setting offered convenience for participants, however it also presented some challenges as it did not always feel very quiet or confidential to some. Additionally, while the shelters themselves were smoke-free and alcohol-free environments, the social pressure, direct or indirect, from fellow shelter residents was challenging. Despite this, participants described a range of motivators and expectations. Many were motivated to enroll for health reasons, including fear of future diagnoses such as cancer. Participants also described the belief that personal willpower was needed before being ready to engage with help and attempt to quit.

*“First of all, change has to come from within; if you’re not ready to change, you’re not going to change. I got irons in every fire I can. My motto is ‘I need all the help I can get!’”(Intervention group participant).*

### Implementation process

Participants were mostly positive about their study participation, and many reported feeling motivated to address their smoking. Participation was described as helping foster sober social time, positive feelings about contributing to the community, and a focused attitude to improve their situation.

*“I think it’s good. It made me feel like I had something to do or like I had a purpose. You know what I mean, not a purpose but it wasn’t like the homeless” (Intervention group participant).*

Participants were able to participate in the intervention activities successfully and for some, the contact with study staff was appreciated. Some reported that they wished there were more cessation counselling sessions available, particularly if they enjoyed the supportive encounters with the study staff. Some participants became champions of the intervention, encouraging other shelter residents to consider enrolling in the study. For some, the study was a welcome activity that focused on their wellness and helped beat experiences of boredom.

Across conditions, participants completed regular study outcome surveys at multiple time points throughout the trial. While a few participants viewed the survey with no particular value, the majority, including control arm participants, viewed this component of the study as meaningful, and helpful in monitoring and reducing smoking behaviors. This suggests that self-monitoring may play an important role in cessation, even if not an intended consequence of the frequent surveys. Notably, nearly all participants were grateful and enthused by the financial incentives.

## Discussion

In this paper, we have applied an implementation science framework, CFIR, to the analysis of the experience of participants in PTQ2 in order to enhance learning on how to best deliver smoking cessation and alcohol abstinence interventions to this at risk population. The outer setting in which the intervention was delivered presented unique challenges for study participants. In particular, the culture of alcohol use and cigarette smoking around the shelter environment presented a serious challenge. There may be a need to consider the impact of broader smoke free policies around shelters. These challenges have previously been reported in the literature [8, 21, 22]. Future research that is responsive to the policy context, or even tests the impact of various policies, would be worthwhile. It has also been reported that the daily life challenges facing people experiencing homelessness can negatively impact smoking cessation [7, 9, 10]; however, participants also described experiencing reduction or cessation as being a helpful strategy to help support broader goals surrounding attainment of permanent housing.

The inner setting of the intervention delivery also emerged as important. The inner setting of the shelters themselves offered a very convenient way to recruit smokers experiencing homelessness. However, this busy, chaotic setting, also proved challenging. As study participants moved away from the shelter, the convenience of the shelter setting transformed into a barrier. It was challenging for participants to return to the shelter, and doing so could expose individuals to pro-smoking and drinking behaviors. Stable housing has been associated with positive outcomes for smoking cessation [23], and finding a way to move the intervention with participants when they move away from the shelter, may be helpful. Potential solutions to these challenges could include ensuring a flexible intervention delivery design, where alternative settings (away from the shelter), or modality (such as phone counselling) are options for cessation counselling.

These was a discrepancy between the goals of the study, cessation, and the goals of individual participants, who felt that reduction was a worthwhile and significant achievement. This poses a challenge for the ways to best address smoking in this community and suggest the need for a broader consideration of the role of reduction in circumstances where there are significant barriers to overcome in the outer setting of the intervention. Future studies should continue to collect data on reduction alongside cessation, and where possible, follow participants long-term so there can be consideration of the long-term benefits of reduction and how it may support future cessation (alongside other factors, such as stabilized housing). Additionally, participants reported that they gained value from the frequent surveys of their health, including of their cigarette and alcohol use, even for those participants who were in the control arm.

### Limitations

This study has some limitations. The sample size is small and may not be representative. Additionally, study participants who felt they were successful in the study may have been more inclined to agree to participate in an interview, and may have overrepresented positive study experiences. Finally, this study specifically recruited smokers experiencing homelessness who also had alcohol use disorder, which may limit the generalizability of the findings beyond this particular group.

## Conclusion

Overall, PTQ2 was well received by study participants, reinforcing the value of continuing to test and offer smoking cessation interventions for people experiencing homelessness. The CFIR framework [14] was useful in offering specific insights about the implementation context of the intervention. Participants described a discord in their personal goals of reduction compared with the study goals of complete abstinence, which may pose a challenge to the ways in which success is defined for people experiencing homelessness.

## Supplementary Information

**Additional file 1.**

## Data Availability

The datasets used and/or analyzed during the current study are available from the corresponding author on reasonable request.

## Abbreviations

CFIR: Consolidated Framework for Implementation Research
PTQ2: Power to Quit 2
