# Supplementary material for: The implementation of a smoking cessation and alcohol abstinence intervention for people experiencing homelessness
Source: BMC Public Health. 2022 Jun 27;22:1260. doi: 10.1186/s12889-022-13563-5 (PMC9235189; doi:10.1186/s12889-022-13563-5)
Supplement: Supplementary file 1 — Additional file 1. [file 12889_2022_13563_MOESM1_ESM.docx]

| *Semi-structured Interview Guide* |
| --- |
| 1. Can you describe to me what got you interested taking part in the PTQ II study? |
| 1. What were you hoping to get out of taking part in the study? |
| 1. What kinds of activities were you doing in the study? |
| 1. You mentioned you received (education/sessions on smoking/sessions on smoking and alcohol) as part of the study. What was your overall impression of doing these activities? |
| 1. How did you feel about the amount of education or counselling you received? |
| 1. Did you use the patch/gum/lozenge? If you didn't use them every day for 12 weeks, what were some of the things that kept you from doing so? |
| 1. Were there any parts of the study activities you particularly liked? |
| 1. Were there any parts of the study activities you particularly did not like? |
| 1. How was it hard, or easy, to make it along to counselling sessions? |
| 1. How would you have felt about it if you had been offered an opportunity to do the counselling by phone? |
| 1. Did the sessions have any impact on your (smoking or smoking and drinking)? |
| 1. What could have made the sessions even better for you? |
| 1. In general, do you have any views on how dealing with homelessness impacts the ability of people to take part in studies like this? |
| 1. Did the counselling sessions have any impact on other aspects of your life, aside from the smoking/smoking and drinking? |
| 1. What did you think about the study questionnaires you were asked to complete? |
| 1. What did you think about the incentives you received? |
| 1. Finally what other topics do you think we should research to help us address smoking for people experiencing homelessness? |

*Semi-structured interview guide*
